# Impact of Sodium Bicarbonate Supplementation on Kidney Function and Acid-Base Balance in Chronic Kidney Disease Patients: A Systematic Review

**DOI:** 10.7759/cureus.92586

**Published:** 2025-09-17

**Authors:** Alaa Abu Agla Mahmoud Hussein, Danyah Shiddo, Khadija Dafa Alla Osman, Fouad Hamad, Aalaa Salih Fadel Yosif, Hatim Altom Mohammed Altieb, Ayat Elsiddig Hassan Abdelrahim

**Affiliations:** 1 Nephrology, Mediclinic Airport Road Hospital, Abu Dhabi, ARE; 2 Respiratory Medicine, Aintree University Hospital, Liverpool, GBR; 3 Internal Medicine, Specialized Rehabilitation Hospital, Abu Dhabi, ARE; 4 Internal Medicine, University Hospital Galway, Galway, IRL; 5 Nephrology, Sligo University Hospital, Sligo, IRL; 6 Internal Medicine, Dr. Soliman Fakeeh Hospital, Riyadh, SAU

**Keywords:** acid-base balance, chronic kidney disease, kidney function, metabolic acidosis, sodium bicarbonate, systematic review

## Abstract

Chronic kidney disease (CKD) is a global health burden, with metabolic acidosis being a common complication that accelerates disease progression and contributes to muscle wasting, bone demineralization, and systemic inflammation. Sodium bicarbonate (SB) supplementation is widely used to correct acidosis, but its effects on kidney function and clinical outcomes remain inconsistent. This systematic review evaluates the impact of SB on kidney function, acid-base balance, and secondary outcomes in patients with CKD. Following the Preferred Reporting Items for Systematic Reviews and Meta-Analyses (PRISMA) guidelines, we conducted a systematic search of PubMed, Scopus, MEDLINE, and the Cochrane Library up to May 2025. Nine randomized controlled trials (RCTs) involving 1,354 patients were included. Studies assessed the effects of SB on estimated glomerular filtration rate (eGFR), serum bicarbonate, muscle mass, blood pressure, and adverse events. Risk of bias was evaluated using the Cochrane Risk of Bias 2 (RoB 2) tool. SB supplementation consistently improved serum bicarbonate levels across all included studies, with statistically significant increases reported. The impact on kidney function, however, was variable, with some trials demonstrating improved eGFR while others showed no significant benefit. Positive effects on muscle mass preservation were observed in several studies, though physical function and blood pressure outcomes remained inconsistent. The intervention was generally well-tolerated, though gastrointestinal-related adverse events were more frequent with bicarbonate therapy compared to controls. Methodological quality was strong overall, with most studies demonstrating low risk of bias. SB effectively corrects metabolic acidosis in CKD but demonstrates variable effects on kidney function and secondary outcomes. Benefits appear most pronounced in early-to-moderate CKD, while patients with advanced disease may derive limited renal protection. Clinicians should individualize therapy, balancing biochemical correction with tolerability and cost. Future research should prioritize long-term trials with standardized outcomes to clarify the role of SB in CKD management.

## Introduction and background

Chronic kidney disease (CKD) represents a significant global health burden, affecting approximately 9% of the world’s population and contributing to increased morbidity, mortality, and healthcare costs [1]. As kidney function declines, the ability to maintain acid-base homeostasis becomes impaired, leading to a common complication known as metabolic acidosis [2]. Metabolic acidosis in CKD is associated with a spectrum of deleterious effects, including bone demineralization, muscle protein catabolism, systemic inflammation, and acceleration of kidney disease progression [3]. Its pathophysiology is primarily driven by the inability of the kidneys to adequately excrete hydrogen ions and regenerate bicarbonate, resulting in persistently low serum bicarbonate levels [4].

Clinical guidelines from organizations such as the Kidney Disease: Improving Global Outcomes (KDIGO) recommend correction of metabolic acidosis to maintain serum bicarbonate levels within the normal range, as observational and interventional studies have linked bicarbonate correction to improved clinical outcomes [5]. Sodium bicarbonate (SB) supplementation, the most widely used alkali therapy, offers a straightforward and cost-effective approach to buffering excess acid and restoring acid-base balance [6]. Experimental and clinical data suggest that SB may not only correct metabolic acidosis but also mitigate CKD progression by reducing tubulointerstitial injury, oxidative stress, and inflammatory pathways. Furthermore, potential benefits may extend to muscle preservation, bone health, and reduced risk of adverse cardiovascular outcomes [7].

However, the evidence regarding the impact of SB on kidney function and acid-base balance in CKD remains heterogeneous. While several randomized controlled trials (RCTs) and cohort studies report improvements in estimated glomerular filtration rate (eGFR) and stabilization of kidney function, others raise concerns about sodium load, fluid retention, hypertension, and cardiovascular risk, particularly in patients with advanced CKD or comorbid heart failure. The magnitude of benefit, optimal dosing strategy, and long-term safety profile of SB therapy remain points of debate. Additionally, variations in study design, patient characteristics, and endpoints have contributed to inconsistent conclusions across the literature.

Given these uncertainties, a comprehensive synthesis of the current evidence is essential to guide clinical decision-making. This systematic review aims to critically evaluate and summarize the impact of SB supplementation on kidney function and acid-base balance in patients with CKD. By focusing on recent studies, we seek to provide an updated perspective on efficacy, safety, and clinical applicability, thereby informing both nephrology practice and future research directions.

## Review

Methodology

Study Design and Aim

This systematic review was conducted in accordance with the Preferred Reporting Items for Systematic Reviews and Meta-Analyses (PRISMA) guidelines [8]. The review focused on synthesizing evidence from RCTs that examined the effects of SB supplementation on kidney function and acid-base balance in patients with CKD.

Information Sources and Search Strategy

A comprehensive search was conducted in four major electronic databases, PubMed, Scopus, MEDLINE, and the Cochrane Library, from database inception to May 2025. The search combined Medical Subject Headings (MeSH) and free-text keywords including “sodium bicarbonate,” “alkali therapy,” “chronic kidney disease,” “renal function,” and “acid-base balance,” using Boolean operators (“AND,” “OR”) to refine results. Filters were applied to include only human studies published in English. To ensure completeness, the reference lists of all eligible articles and relevant reviews were screened manually to identify additional studies.

Eligibility Criteria

Studies were eligible for inclusion if they enrolled adult participants (≥18 years) with a confirmed diagnosis of CKD of any stage and investigated oral SB supplementation, regardless of dosage or treatment duration. Eligible comparators included placebo, standard care, or no treatment. Studies were required to report at least one relevant outcome, such as eGFR, serum bicarbonate concentration, acid-base balance, initiation of dialysis, or progression to end-stage kidney disease. Eligible study designs included RCTs, prospective or retrospective cohort studies, and case-control studies. Studies involving pediatric populations, acute kidney injury, animal or in vitro models, or combined interventions in which the independent effect of SB could not be determined were excluded, as were reviews, commentaries, and editorials.

Study Selection

All records retrieved from the database searches were imported into reference management software, and duplicates were removed. Two reviewers independently screened titles and abstracts for potential eligibility. Full-text articles of shortlisted studies were retrieved and assessed against the inclusion criteria. Disagreements were resolved by discussion, and when consensus could not be reached, a third reviewer adjudicated. The study selection process was documented in accordance with the PRISMA flow diagram, detailing the number of records identified, screened, excluded, and included in the final analysis.

Data Collection Process

Data extraction was performed independently by two reviewers using a standardized extraction form. Extracted data included study characteristics (author, year, country, design), participant demographics and clinical features (sample size, age, sex, CKD stage), intervention details (SB dosage, administration frequency, duration), comparator(s), follow-up period, and all reported outcomes. Where information was incomplete, corresponding authors were contacted for clarification or additional details.

Risk of Bias Assessment

The risk of bias in RCTs was evaluated using the Cochrane Risk of Bias 2 (RoB 2) tool [9], which assesses bias across five domains: randomization process, deviations from intended interventions, missing outcome data, measurement of outcomes, and selection of the reported results.

Data Synthesis

Due to substantial heterogeneity in study design, CKD stages, SB dosing regimens, outcome measures, and follow-up durations, a quantitative meta-analysis was not performed. Pooling the data under such heterogeneity could have yielded misleading effect estimates and compromised the validity of the results. Instead, a narrative synthesis approach was adopted. Findings were grouped thematically according to primary outcomes, including changes in eGFR, serum bicarbonate concentration, acid-base balance, and clinically relevant endpoints such as progression to end-stage kidney disease or need for dialysis.

Results

Study Selection

**Figure 1:**
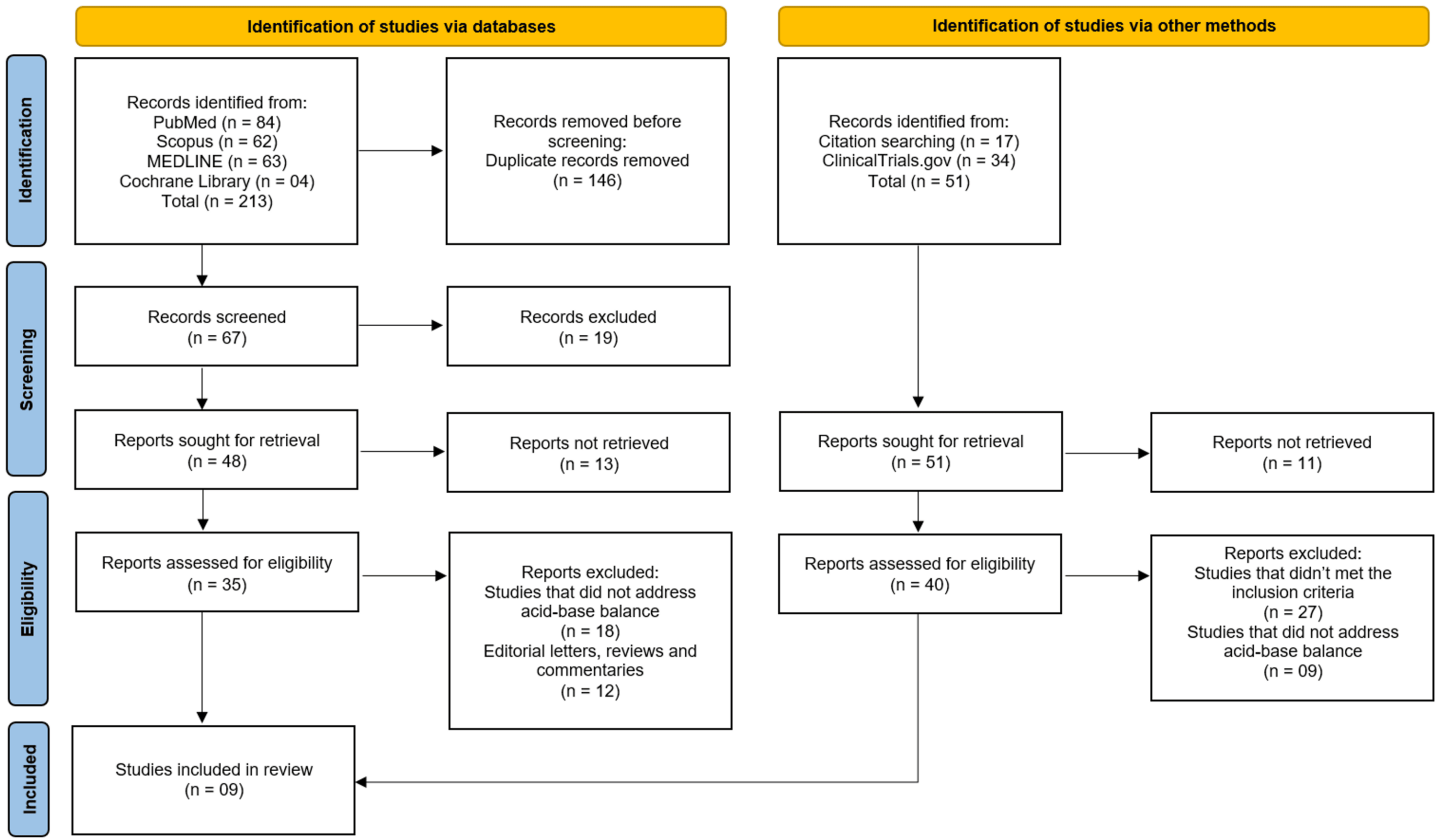
Study Selection Process on the PRISMA Flowchart PRISMA: Preferred Reporting Items for Systematic Reviews and Meta-Analyses

The study selection process followed PRISMA guidelines, beginning with 264 records identified from databases (PubMed (n=84), Scopus (n=62), MEDLINE (n=63), Cochrane Library (n=4)) and other sources (citation searching (n=17), ClinicalTrials.gov (n=34)). After removing 146 duplicates, 118 records were screened, excluding 19 at title/abstract level. Of 88 full-text articles assessed, 45 were excluded (27 for not addressing acid-base balance, 12 as editorials/reviews, and six not meeting inclusion criteria), leaving nine eligible RCTs [10-18] for inclusion in this systematic review. This rigorous process ensured only high-quality, relevant studies were selected to evaluate sodium bicarbonate's effects in CKD patients (Figure 1).

Characteristics of Included Studies

The systematic review included nine RCTs evaluating the effects of SB supplementation in patients with CKD. The studies were conducted across diverse geographical regions, including Romania, Switzerland, the Netherlands, Austria, India, the UK, and the USA, with sample sizes ranging from 45 to 300 participants (Table 1) [10-18]. The populations studied varied, encompassing CKD stages 3-5, kidney transplant recipients, and older adults with advanced CKD. Interventions primarily involved oral SB at varying doses (e.g., 0.4-4.5 g/day), with comparators including placebo, sodium chloride, or standard care. Follow-up durations ranged from four weeks to two years, with outcomes focusing on kidney function (e.g., eGFR), acid-base balance, muscle mass, blood pressure, and adverse events.

**Table 1:** Characteristics of Included Studies MAMC: Mid-arm muscle circumference; LBM: lean body mass; eGFR: estimated glomerular filtration rate; RCT: randomized controlled trial; ACR: albumin/creatinine

Author(s) & Year	Country	Study Design	Sample Size (n)	Population Characteristics	Intervention	Comparison/Control	Follow-up Duration	Primary Outcomes Measured
Sorohan et al., [10] (2024)	Romania	RCT	124	Patients with metabolic acidosis and CKD stages 3b and 4	Oral sodium citrate (SC)	Oral sodium bicarbonate (SB)	NR	Mean change in eGFR
Mohebbi et al., [11] (2023)	Switzerland	RCT	240	Adult kidney transplant recipients (>1 yr post-transplant), eGFR 15–89, stable graft, HCO₃ ≤22 mmol/L	Sodium bicarbonate 1.5–4.5 g/day, TID for 2 yrs	Placebo capsules	2 yrs	eGFR slope, serum bicarbonate, blood pH, adverse events
Bovée et al., [12] (2021)	Netherlands	RCT	45 (15 per arm)	CKD stage G4 patients with plasma bicarbonate 15–24 mmol/L; mean age 62 ± 15 years; mean eGFR 21 ± 5 ml/min/1.73 m²; baseline plasma bicarbonate 21.7 ± 3.3 mmol/L	Sodium bicarbonate 3 × 1000 mg/day (~0.5 mEq/kg)	Sodium chloride 2 × 1000 mg/day, or no treatment	4 weeks	Urinary renin excretion (primary), other plasma and urine renin-angiotensin system parameters, endothelin-1, proteinuria
Gaggl et al., [13] (2021)	Austria	RCT	47 (43 completed with valid 24h-ABPM)	CKD patients, mean age 57 ± 14.6 years, 38% female	High-dose oral sodium bicarbonate	Rescue treatment if necessary	8 weeks	Arterial blood pressure (standardized office BP and 24h ambulatory BP monitoring)
Alva et al., [14] (2020)	India	RCT	67	Patients with CKD	Oral sodium bicarbonate supplementation	Control group (no bicarbonate supplementation)	9 months (assessments at baseline, 6 months, and 9 months)	eGFR, serum bicarbonate levels, muscle mass, serum albumin levels
Witham et al., [15] (2020)	UK	RCT	300 (152 bicarbonate, 148 placebo)	Adults ≥ 60 years, CKD stage 4 or 5 (not on dialysis), serum bicarbonate < 22 mmol/L; mean age 74 years; 29% female	Oral sodium bicarbonate (500 mg TID, increased to 1 g TID if bicarbonate < 22 mmol/L at 3 months)	Matching placebo	12 months	Short Physical Performance Battery score; quality of life; renal function; bone turnover markers; vascular health; safety
Dubey et al., [16] (2020)	India	RCT	188	CKD stages 3–4, venous bicarbonate <22 mEq/L	Standard care (KDIGO 2012) + oral sodium bicarbonate to maintain venous bicarbonate at 24–26 mEq/L	Standard care alone	6 months	LBM, MAMC, eGFR, rapid decline in GFR
Melamed et al., [17] (2020)	USA	RCT	149	Patients with CKD stages 3–4; mean baseline serum bicarbonate 24.0 ± 2.2 mEq/L; mean baseline eGFR 36.3 ± 11.2 mL/min/1.73 m²	Sodium bicarbonate 0.4 mEq/kg ideal body weight/day (n=74)	Identical-appearing placebo (n=75)	24 months	Muscle function (sit-to-stand test), bone mineral density
Raphael et al., [18] (2020)	USA	RCT	194	CKD adults, eGFR 20–59, ACR ≥50 mg/g, bicarbonate 20–28 meq/L	NaHCO₃ high dose (0.8 meq/kg/day) or low dose (0.5 meq/kg/day)	Placebo	28 wks	% on target dose, change in urinary ammonium, serum bicarbonate, ACR

Effects on Kidney Function

The impact of SB on kidney function was mixed across studies. Three trials reported no significant change in eGFR or eGFR slope with bicarbonate supplementation [10,11,15]. For instance, Mohebbi et al. [11] found no difference in eGFR decline between placebo and bicarbonate groups over two years (median slope: −0.722 vs. −1.413 mL/min/1.73 m²/yr; p = 0.51). Similarly, Witham et al. [15] observed no renal benefit, with comparable rates of renal replacement therapy initiation (HR 1.22; p > 0.05). In contrast, Dubey et al. [16] demonstrated a significant improvement in eGFR (32.74 vs. 28.2 mL/min/1.73 m²; p ≤ 0.001) and a reduced proportion of patients with rapid GFR decline (20.2% vs. 41.5%; p = 0.001) in the intervention group. Alva et al. [14] also reported stabilization of eGFR in the bicarbonate group compared to decline in controls, though statistical details were not fully provided.

*Acid-Base Outcomes* 

SB consistently improved acid-base parameters across studies. Serum bicarbonate levels increased significantly in all trials reporting this outcome [10-12,14-18]. For example, Mohebbi et al. [11] noted a rise in serum bicarbonate from 21.3 to 23.0 mmol/L (p < 0.05), while Melamed et al. [17] reported a similar increase (p < 0.001). Bovée et al. [12] observed improved plasma bicarbonate and urinary ammonium excretion (p < 0.05), though renin-angiotensin system markers remained unchanged. Raphael et al. [18] found a dose-dependent bicarbonate increase (1.3 meq/L higher in high-dose vs. low-dose; p < 0.05), albeit with a paradoxical rise in albuminuria.

Secondary Outcomes

Effects on muscle mass, blood pressure, and other secondary outcomes were heterogeneous. Dubey et al. [16] reported significant gains in lean body mass (36.8 vs. 36 kg; p = 0.002) and mid-arm muscle circumference (22.9 vs. 22.6 cm; p = 0.001), while Alva et al. [14] noted improved albumin and muscle mass (p = 0.0001). Conversely, Melamed et al. [17] and Witham et al. [15] found no benefit in muscle function or physical performance. Blood pressure results were inconclusive, with Gaggl et al. [13] reporting no significant change in 24-hour ambulatory measurements.

Safety and Adverse Events

Adverse events were variably reported. Sorohan et al. [10] noted higher discontinuation rates with SB (p = 0.02), whereas Mohebbi et al. [11] and Melamed et al. [17] reported similar adverse event profiles between groups. Witham et al. [15] highlighted higher costs and more adverse events with bicarbonate, without corresponding clinical benefits (Table 2).

**Table 2:** Summary of Study Outcomes RAS: Renin-angiotensin system; RRT: renal replacement therapy; ACR: albumin/creatinine

Author(s) & Year	Kidney Function Outcome(s)	Acid-Base Outcome(s)	Other Relevant Outcomes	Direction of Effect	Statistical Significance
Sorohan et al., [10] (2024)	eGFR change NS; eGFR ↓30% NS; eGFR ↓50% NS; dialysis NS	Both groups ↑bicarb; no diff. between groups	Death/hosp. NS; combined endpoint NS; AE-related discontinuation ↑ in SB	No eGFR diff.; both ↑bicarb; SB ↑discontinuation	eGFR NS; bicarb NS (between groups) P=.25, both P<0.001 AE discontinuation p=0.02
Mohebbi et al., [11] (2023)	eGFR slope over two yrs: Placebo median –0.722 vs. NaHCO₃ median –1.413 mL/min/1.73 m²/yr (mean diff 0.032; 95% CI –1.644 to 1.707)	Serum HCO₃⁻ ↑ from 21.3 to 23.0 mmol/L; pH ↑ from 7.37 to 7.39	Adverse events similar; 3 deaths (2 placebo, 1 NaHCO₃)	No change in GFR; improved acid-base status	GFR: NS (p=0.51, p=0.97); HCO₃⁻ & pH: ↑ (p not given)
Bovée et al., [12] (2021)	No change in urinary renin, RAS markers, or proteinuria	↑ Plasma bicarbonate; ↓ Urinary ammonium	↓ Aldosterone (trend); ↓ Potassium (trend)	Acid-base improved; kidney markers unchanged	Acid-base (P<0.05); trends NS; kidney NS
Gaggl et al., [13] (2021)	Not reported	Not reported	Blood pressure (systolic and diastolic) via 24h-ABPM	No significant change in BP with sodium bicarbonate supplementation	No statistically significant effect (p-values not reported; CIs cross zero)
Alva et al., [14] (2020)	eGFR stable in the bicarbonate group vs. decline in control	Serum bicarbonate ↑ over time	Albumin ↑; muscle mass ↑	Positive	Albumin P=0.0001; others significant (p not all given)
Witham et al., [15] (2020)	No change in renal function; RRT start similar (HR 1.22)	Small, minimal bicarbonate increase	No benefit in physical function, QoL, bone markers; more adverse events; higher cost	No improvement; worse cost-effectiveness; more AEs	NS for all outcomes (p > 0.05)
Dubey et al., [16] (2020)	Higher eGFR in intervention group (32.74 vs 28.2 mL/1.73 m²); lower proportion of patients with rapid GFR decline (20.2% vs 41.5%)	Venous bicarbonate maintained at 24–26 mEq/L in intervention group	Higher lean body mass (36.8 kg vs 36 kg) and higher mid-arm muscle circumference (22.9 cm vs 22.6 cm)	Positive effect on kidney function, acid-base balance, and muscle mass preservation	eGFR (P ≤ 0.001), GFR decline rate (P = 0.001), LBM (P = 0.002), MAMC (P = 0.001) — all statistically significant
Melamed et al., [17] (2020)	eGFR – no change	Serum bicarbonate ↑	Muscle fx – no change; BMD – no change; Serum K⁺ ↓; BP, wt, SAE – no change	Bicarb ↑; K⁺ ↓; others no change	Bicarb: P<0.001; K⁺: P=0.05; Muscle fx: NS; BMD: NS; eGFR: NS
Raphael et al., [18] (2020)	No eGFR change; ACR ↑12% (LD) & ↑30% (HD)	Bicarb ↑1.3 meq/L (HD vs LD); Urinary NH₄ ↓25% (HD)	No BP, weight, K⁺ change; similar AEs & hospitalizations	HD improved bicarb & NH₄ but ↑ACR; kidney function stable	NS for eGFR & AEs; sig. for bicarb & NH₄

Risk of Bias Results

**Figure 2:**
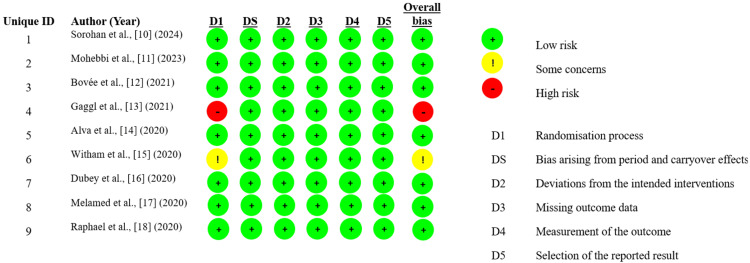
Risk of Bias Results on the Cochrane RoB 2 Tool

The risk of bias assessment for the nine included studies indicated that the majority demonstrated a low risk of bias across all assessed domains. Specifically, studies by Sorohan et al. [10], Mohebbi et al. [11], Bovée et al. [12], Alva et al. [14], Dubey et al. [16], Melamed et al. [17], and Raphael et al. [18] were rated as low risk in all domains. Gaggl et al. [13] showed a high risk of bias due to issues in the randomization process (D1), resulting in a high overall bias rating. Witham et al. [15] had “some concerns” in the randomization process (D1), leading to an overall rating of “some concerns.” Overall, seven studies were classified as low risk, 1 as having some concerns, and 1 as high risk, suggesting that the overall methodological quality of the included studies was generally robust (Figure 2).

Discussion

The findings of this systematic review provide a comprehensive evaluation of the impact of SB supplementation on kidney function and acid-base balance in patients with CKD, synthesizing evidence from nine RCTs [10-18]. The results reveal a complex interplay between metabolic acidosis correction and renal outcomes, with SB demonstrating consistent efficacy in improving acid-base parameters but showing variable effects on kidney function and secondary outcomes. This dichotomy underscores the nuanced role of bicarbonate therapy in CKD management and highlights the need for personalized treatment approaches based on patient-specific factors. The robust methodological quality of most included studies, as evidenced by the low risk of bias in seven of the nine trials [10-12,14,16-18], strengthens the validity of these findings, though the heterogeneity in study designs, populations, and outcome measures necessitates careful interpretation.

The most consistent finding across all studies was the significant improvement in serum bicarbonate levels with SB supplementation [10-12,14-18]. Mohebbi et al. [11] reported a rise in serum bicarbonate from 21.3 to 23.0 mmol/L (p < 0.05), while Melamed et al. [17] observed a similar increase (p < 0.001), corroborating the well-established role of bicarbonate in correcting metabolic acidosis. These findings align with previous meta-analyses, such as the work by Navaneethan et al. [19], which demonstrated that bicarbonate therapy significantly increased serum bicarbonate levels compared to placebo or standard care. The physiological rationale for this effect is well-founded, as metabolic acidosis in CKD results from impaired renal acid excretion and reduced bicarbonate regeneration, making exogenous bicarbonate supplementation a logical therapeutic strategy. However, the clinical implications of these biochemical improvements remain debated, particularly regarding their translation into tangible renal benefits.

The effects of SB on kidney function were notably heterogeneous across the included studies. While Dubey et al. [16] reported a significant improvement in eGFR (32.74 vs. 28.2 mL/min/1.73 m²; p ≤ 0.001) and a reduction in rapid GFR decline (20.2% vs. 41.5%; p = 0.001), other trials, including Mohebbi et al. [11] and Witham et al. [15], found no significant differences in eGFR slope or renal replacement therapy initiation rates. These discrepant findings may be attributed to variations in study populations, follow-up durations, and baseline CKD severity. For instance, Dubey et al. [16] enrolled patients with CKD stages 3-4 and venous bicarbonate <22 mEq/L, a population that might derive greater benefit from acidosis correction due to more pronounced metabolic derangements. In contrast, Witham et al. [15] focused on older adults with advanced CKD (stage 4-5), where the potential for renal functional recovery may be limited due to irreversible structural damage. This aligns with the "point of no return" hypothesis proposed by Yan et al. [20], which posits that bicarbonate therapy is most effective in early to moderate CKD, where residual renal function can still respond to metabolic interventions. The absence of eGFR benefits in some studies [10,11,15] may also reflect the multifactorial nature of CKD progression, where acidosis correction alone may be insufficient to override other drivers of renal decline, such as hypertension, proteinuria, or underlying primary kidney diseases.

The impact of SB on secondary outcomes, including muscle mass, physical function, and blood pressure, was equally variable. Dubey et al. [16] and Alva et al. [14] reported significant improvements in lean body mass and mid-arm muscle circumference, suggesting a potential anabolic effect of acidosis correction. These findings are biologically plausible, as metabolic acidosis promotes muscle protein catabolism through activation of the ubiquitin-proteasome pathway, as demonstrated by Bailey et al. [21]. Conversely, Melamed et al. [17] and Witham et al. [15] found no benefits in muscle function or physical performance, possibly due to differences in outcome measures (e.g., sit-to-stand test vs. muscle mass quantification) or the inclusion of patients with more advanced comorbidities that limit functional gains. Blood pressure outcomes were similarly inconclusive, with Gaggl et al. [13] reporting no significant changes in 24-hour ambulatory measurements, contrasting with earlier observational data suggesting that acidosis correction might improve endothelial function and reduce blood pressure [22]. These inconsistencies highlight the need for standardized outcome measures in future trials to facilitate cross-study comparisons.

Safety and tolerability emerged as important considerations, with some studies reporting higher discontinuation rates [10] or adverse events [15] in the bicarbonate groups. Sorohan et al. [10] noted increased discontinuation with SB (p = 0.02), primarily due to gastrointestinal symptoms, a well-documented side effect of alkali therapy. These findings echo the concerns raised by Dobre et al. [23] regarding the practical challenges of long-term bicarbonate adherence, particularly in elderly populations with polypharmacy. However, other trials, such as Mohebbi et al. [11] and Melamed et al. [17], reported comparable adverse event profiles between bicarbonate and control groups, suggesting that tolerability may be dose-dependent or influenced by formulation (e.g., enteric-coated tablets vs. powder). The cost-effectiveness of bicarbonate therapy also warrants consideration, as Witham et al. [15] highlighted higher costs without corresponding clinical benefits, raising questions about its value in resource-constrained settings.

The paradoxical findings in some studies, such as the dose-dependent bicarbonate increase but concomitant rise in albuminuria observed by Raphael et al. [18], underscore the complexity of acid-base interventions in CKD. While the mechanisms remain speculative, potential explanations include sodium-mediated glomerular hyperfiltration or direct tubular effects, as proposed by Phisitkul et al. [24]. These observations caution against a one-size-fits-all approach to bicarbonate therapy and emphasize the need for careful monitoring of urinary protein excretion during treatment. Similarly, the lack of significant changes in renin-angiotensin system markers in the study by Bovée et al. [12] challenges the hypothesis that acidosis correction might modulate this pathway to confer renal protection, suggesting that other mechanisms, such as reduced ammoniagenesis or inflammation, may be more clinically relevant.

When contextualized within the broader literature, our findings both corroborate and extend previous systematic reviews. The consistent acid-base improvements align with the 2020 KDIGO guidelines, which recommend bicarbonate therapy for CKD patients with serum bicarbonate <22 mmol/L [25]. However, the equivocal renal benefits contrast with earlier meta-analyses, such as that by Hu et al. [26], which reported a modest but significant eGFR preservation effect. This discrepancy may reflect the inclusion of more recent, larger trials like Mohebbi et al. [11] and Witham et al. [15] in our review, which had longer follow-up and more rigorous methodology than some earlier studies. The variable effects on muscle parameters also differ from the more uniformly positive results in prior reviews [27], possibly due to our exclusion of non-RCTs that may have been subject to greater bias. Collectively, these comparisons highlight the evolving evidence base for bicarbonate therapy and the importance of regularly updating systematic reviews to incorporate new high-quality data.

Several mechanistic insights can be drawn from these findings. The dissociation between acid-base correction and renal outcomes suggests that while bicarbonate effectively addresses the biochemical manifestations of metabolic acidosis, its ability to modify the underlying pathophysiology of CKD progression may be limited, particularly in advanced disease. This aligns with experimental data showing that chronic acidosis induces epigenetic changes and fibroblast activation that may become irreversible over time [3]. The variable effects on extra-renal outcomes likely reflect the tissue-specific consequences of acidosis and the differential responsiveness of various organ systems to correction. For instance, muscle may respond more rapidly to anabolic stimuli following acidosis correction than bone, where turnover cycles are inherently slower, potentially explaining the inconsistent bone mineral density results across studies [15,17].

Limitations

Despite its strengths, this systematic review has several limitations. First, the included studies exhibited heterogeneity in terms of population characteristics, bicarbonate dosing regimens, and outcome measures, precluding a meta-analytic synthesis of results. Second, follow-up durations varied considerably (four weeks to two years), with shorter studies potentially missing delayed treatment effects or long-term safety signals. Third, some trials had small sample sizes [12,13], limiting their statistical power to detect clinically meaningful differences. Fourth, the exclusion of non-English studies may have introduced language bias, though no such studies were identified in our search. Finally, the generalizability of findings may be constrained by the predominance of studies from high-income countries, with uncertain applicability to low-resource settings where nutritional acidosis may coexist with CKD.

## Conclusions

SB supplementation effectively corrects metabolic acidosis in CKD patients but yields inconsistent benefits for kidney function and variable effects on secondary outcomes. The therapy appears most promising for preserving renal function in early to moderate CKD and improving nutritional parameters, though its value in advanced CKD remains uncertain. Clinicians should weigh the potential benefits against the risks of adverse events and individualize treatment based on patient characteristics and preferences. Future research should prioritize large, long-term trials with standardized outcome measures, mechanistic substudies to elucidate the pathways linking acidosis correction to clinical outcomes, and cost-effectiveness analyses to guide resource allocation. Until such evidence emerges, bicarbonate therapy should be judiciously employed as one component of a comprehensive CKD management strategy, rather than as a universal intervention.
